# Mitoxantrone synergies with tigecycline against *Klebsiella pneumoniae*

**DOI:** 10.1128/spectrum.00568-26

**Published:** 2026-08-12

**Authors:** Xin Xia, Xiaoya Wei, Shuiping Chen, Zhihui Cheng, Yongxin Jin, Shouguang Jin, Weihui Wu

**Affiliations:** 1 State Key Laboratory of Medicinal Chemical Biology, Key Laboratory of Molecular Microbiology and Technology of the Ministry of Education, Department of Microbiology, College of Life Sciences, Nankai University 12538, Tianjin, China; 2 Department of Laboratory Medicine, Fifth Medical Center of PLA General Hospital 26460, Beijing, China; National Institute of Science Education and Research, Jatni, Odisha, India

**Keywords:** tigecycline, mitoxantrone, *Klebsiella pneumoniae*

## Abstract

The global health threat of antimicrobial resistance necessitates the development of novel therapeutic strategies. This study identifies the anticancer agent mitoxantrone as a potent antimicrobial against *Klebsiella pneumoniae*. Through passaging experiments, we demonstrate that resistance to mitoxantrone arises via mutations in the *kstR2* gene, leading to the overexpression of the SmvA efflux pump. Transcriptomic analysis reveals that mitoxantrone treatment induces the bacterial SOS response, likely due to DNA damage. Critically, we found that mitoxantrone exhibits a synergistic effect with tigecycline against *K. pneumoniae*, both *in vitro* and in a murine wound infection model. Mechanistically, this synergy is driven by tigecycline-mediated enhancement of intracellular mitoxantrone accumulation. These findings reveal a potentially effective combination regimen to combat *K. pneumoniae* infections.

**IMPORTANCE:**

The rise of antibiotic-resistant *Klebsiella pneumoniae* demands new treatment options. This study uncovers that mitoxantrone, a drug currently approved for cancer therapy, is effective against *K. pneumoniae*. Furthermore, we discovered that combining mitoxantrone with the existing antibiotic tigecycline is synergistic against *K. pneumoniae*. We found that tigecycline enhances mitoxantrone accumulation inside bacterial cells. Overall, our work identified the tigecycline-mitoxantrone combination as a promising strategy to treat *K. pneumoniae* infections.

## INTRODUCTION

Antimicrobial resistance (AMR) is threatening the effectiveness of antibiotics and public health worldwide. AMR endangers medical procedures such as surgery, organ transplantation, chemotherapy, and diabetes management (1). In 2019, 4.95 million deaths were related to antibiotic-resistant bacterial infections, among which 1.27 million were directly caused by antibiotic-resistant bacterial infections (2). If left unaddressed, AMR is predicted to cause 10 million deaths and cost approximately 100 trillion US dollars globally by 2050 (3). To address this challenge, new antibiotics and therapeutic strategies are urgently needed (4–6).

Combination therapy is a common strategy in clinical practice for severe infections (7). Successful combination therapy typically has the following advantages: synergistic effect, inhibition of bacterial resistance development, and reduction of the dosage of a single drug, thereby reducing toxicity (8). The main mechanisms by which non-antibiotic adjuvants produce synergistic effects with antibiotics include efflux pump inhibition, increased membrane permeability, biofilm disruption, enzyme activity inhibition, and immune response modulation (9). Currently, one of the most widely used combined therapies of non-antibiotics and antibiotics is the combination of β-lactamase inhibitors and β-lactam antibiotics, such as ceftazidime-avibactam, piperacillin-tazobactam, and cefoperazone-sulbactam (10).

Drug repurposing is another strategy for developing new antimicrobials and combination therapies from approved or investigational drugs that are outside the scope of the original medical applications (11). The advantages of this strategy include lower failure risk, reduced drug development time, lower investment needs, and potential to reveal new targets and pathways for further exploitation (12–14). Torres et al. found that seven small-molecule drugs (clomiphene citrate, mitoxantrone DHCl, thiostrepton, methyl benzethonium, benzethonium Cl, auranofin, and thonzonium Br) can exert a synergistic effect with polymyxin B to inhibit the growth and survival of planktonic *Pseudomonas aeruginosa* PAO1 and effectively eliminate its biofilm (15). Cai et al. found that the antineoplastic drug daunorubicin significantly enhances the activities of colistin and tigecycline against multidrug-resistant gram-negative bacteria pathogens and biofilm-producing bacteria, effectively inhibiting the evolution and spread of resistance (16).

Traditional minimum inhibitory concentration (MIC) assays are routinely performed in the cation-adjusted Mueller-Hinton broth (CA-MHB) that differs markedly from human infection sites (sputum, blood, urine, etc.). Thus, the *in vitro* results might over- or underestimate the actual *in vivo* efficacies of antibiotics (17). The same limitation applies to screens for non-antibiotic adjuvants: compounds that are active in the host environment may be missed when screening is carried out in CA-MHB or other regular bacterial culture medium, such as Luria-Bertani (LB) (18). It has been demonstrated that antimicrobial activities can be significantly influenced by testing media (19, 20). For instance, *Acinetobacter baumannii* were more susceptible to polymyxins and azithromycin in a physiologically relevant culture medium than in MHB, which was corroborated by the bacteria killing results in murine and *Galleria mellonella* infection models (21, 22). As for *P. aeruginosa*, the MIC of azithromycin is 16-fold lower in a physiologically relevant medium than that in MHB. Results from a murine abscess model verified the effectiveness of azithromycin (23). Therefore, evaluation of antimicrobial activities in physiologically relevant media might facilitate the discovery of effective antimicrobials.

In a previous screen of the Prestwick Chemical Library (1200 compounds), clomiphene, tamoxifen, vanoxerine, terfenadine, mitoxantrone, and amiodarone were found to be effective against *Streptococcus pneumoniae* (24). In this study, we utilized a physiologically relevant medium (23) to evaluate the antimicrobial activities of these compounds against gram-negative bacterial pathogens. Our results demonstrated that mitoxantrone is effective against *K. pneumoniae*. In addition, mitoxantrone synergizes with tigecycline *in vitro* and *in vivo*. Mechanistic studies revealed that tigecycline promotes the accumulation of mitoxantrone.

## RESULTS

### Mitoxantrone is effective against *K. pneumoniae*

To examine antibacterial activities of the six compounds against gram-negative pathogenic bacteria, we performed MIC assays in the physiologically relevant medium using *P. aeruginosa*, *A. baumannii*, and *Klebsiella pneumoniae*. Among the compounds, mitoxantrone (MTX) displayed an MIC of 0.25 mg/L against the *K. pneumoniae* strain Kp4325 (Table 1). To examine the efficacy of MTX against hard-to-treat strains, including carbapenem-resistant clinical isolates (Kp01, Kp03, Kp08, Kp09, Kp10, and Kp43) and a polymyxin B-resistant strain (FYJ945) (25), alongside a reference strain ATCC700603 (Table S1), the MICs were determined in the physiologically relevant medium. Sequence types and capsular types of the clinical isolates are shown in Table S1. The MICs of MTX for the *K. pneumoniae* isolates ranged from 0.5 to 16 mg/L (Table 2), demonstrating the effectiveness of MTX against *K. pneumoniae*. We then investigated the resistance evolution and bacterial response to MTX in *K. pneumoniae*.

**TABLE 1:** MICs (mg/L) of the tested compounds^*a*^

Compound	PA14	AB233	KP4325
Clomiphene citrate	>512	>512	>512
Tamoxifen citrate	>512	>512	>512
Vanoxerine dihydrochloride	>512	512	>512
Terfenadine	>512	>64	>512
Mitoxantrone dihydrochloride	>64	8	0.25
Amiodarone hydrochloride	>512	>512	>512

^*a*^ Data represent results from three independent experiments.

**TABLE 2:** MICs (mg/L) of mitoxantrone (MTX) dihydrochloride against *K. pneumoniae* clinical isolates

Strains^*a*^	MTX
Kp01	8
Kp03	8
Kp08	4
Kp09	0.5
Kp10	2
Kp43	4
FYJ945	8
ATCC 700603	16

^*a*^ Data represent results from three independent experiments.

### Mutations in the *kstR2* gene contributed to the bacterial resistance to MTX

**Fig 1:**
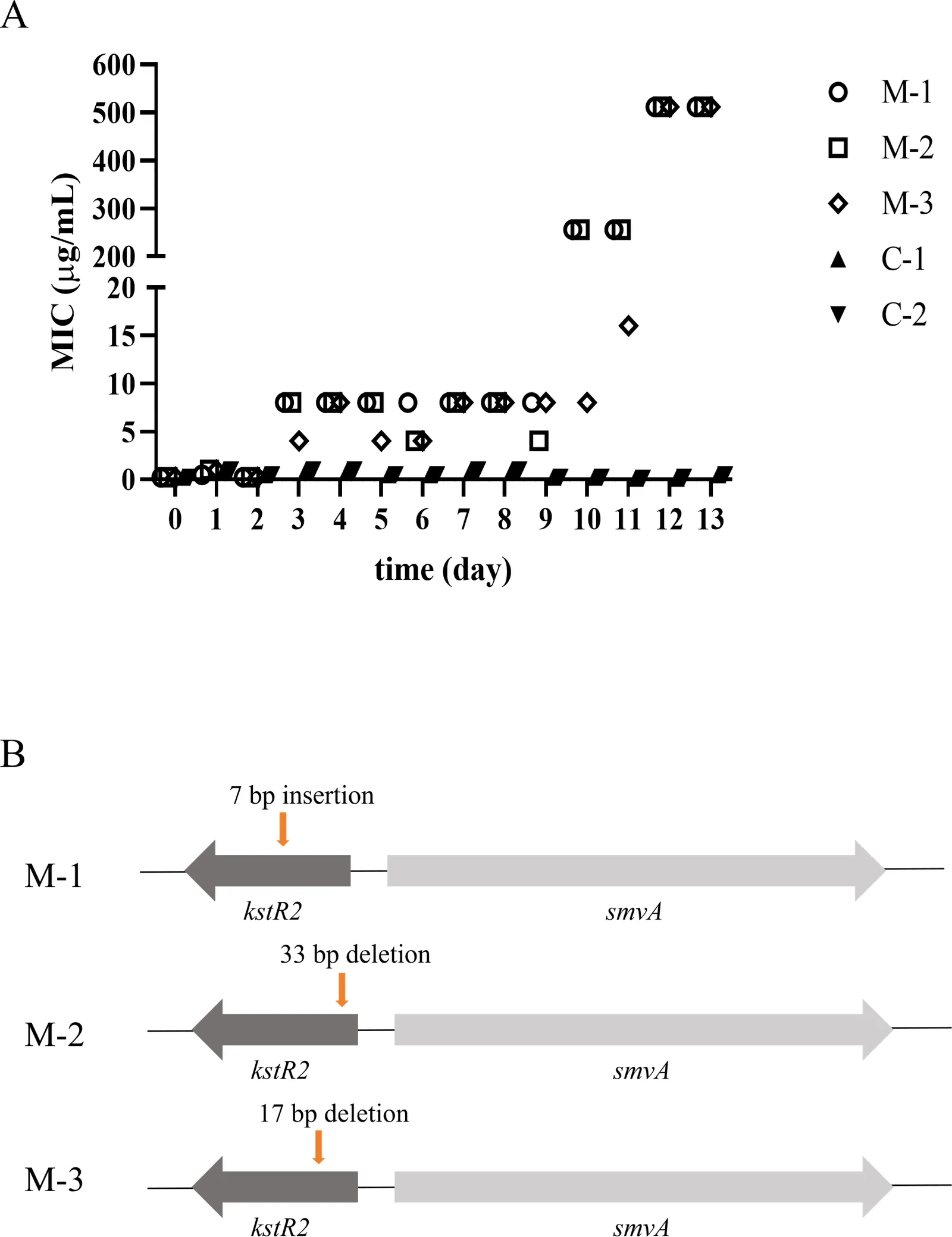
Development of mitoxantrone resistance by Kp4325. (**A**) Dynamics of stepwise resistance development to mitoxantrone in Kp4325. Three parallel repeats were performed in the presence of mitoxantrone (designated M-1 to M-3). Another two parallel repeats were passaged in the physiologically relevant medium (designated C1 and C2). (**B**) Schematic presentation of mutations in the *kstR2* gene in mitoxantrone-resistant mutants.

The development of resistance of Kp4325 to MTX was examined with an *in vitro* passage assay in the physiologically relevant medium with three parallel repeats (Fig. 1A). A 32- to 64-fold increase of MICs occurred at the 10th passage of parallel #1, #2, and the 12th passage of parallel #3, where the MICs reached 256 and 512 mg/L, respectively (Fig. 1A). Single colonies from each of the parallels at the corresponding passages were isolated and designated as M-1, M-2, and M-3. Strains passaged in the physiologically relevant medium were designated as C-1 and C-2. The MICs of M-1, M-2, and M-3 were the same as the corresponding populations (Table 3).

**TABLE 3:** MICs (mg/L) of mitoxantrone dihydrochloride against indicated *K. pneumoniae* isolates

Strains^*a*^	MIC
M-1	256
M-2	256
M-3	512
C-1	0.25
C-2	0.25
M-1/pUCP24	256
M-1/pUCP24-*kstR2*	2
M-2/pUCP24	256
M-2/pUCP24-*kstR2*	2
M-3/pUCP24	512
M-3/pUCP24-*kstR2*	4
M-3Δ*smvA*	4

^*a*^ Data represent results from three independent experiments.

Whole-genome sequencing revealed that all of the three resistant mutants harbor a mutation in a TetR family transcriptional regulator (TFR) gene *kstR2* (Fig. 1B; Table S3). Complementation with a wild-type *kstR2* gene in the three mutants reduced the MICs by 128-fold (Table 3).

**Fig 2:**
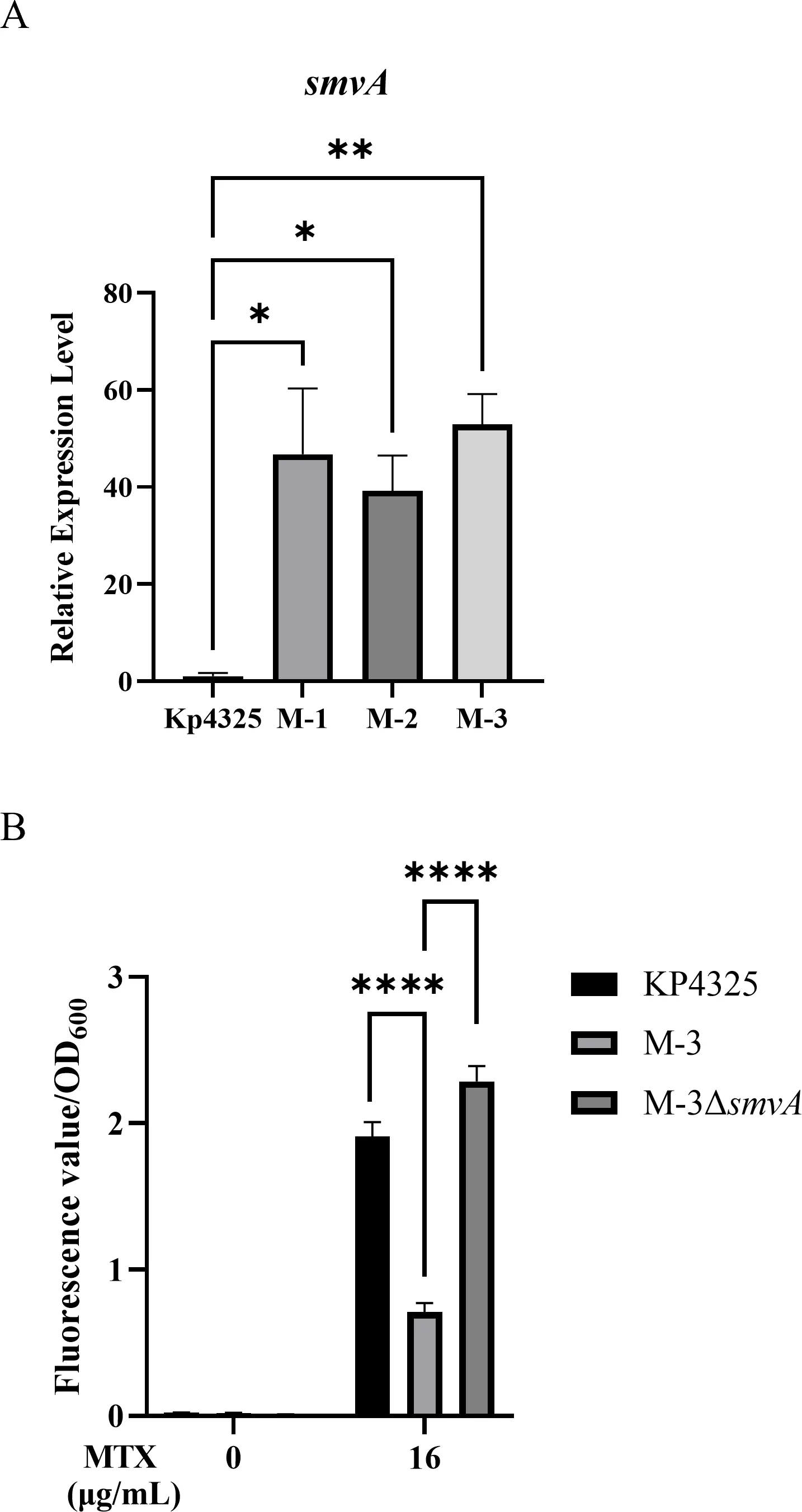
SmvA is involved in the mitoxantrone resistance in the evolved resistant mutant. (**A**) mRNA levels of *smvA* in Kp4325 and the resistant mutants M-1, M-2, and M-3. The *rpoB* gene was used as an internal control. *, *P* < 0.05; **, *P* < 0.01 by one-way ANOVA. The experiment was performed with two technical replicates. Data shown are representative of three independent experiments. (**B**) Intracellular amounts of mitoxantrone in Kp4325, M-3, and M-3∆*smvA*. The relative intracellular accumulation of mitoxantrone was determined after incubation with 16 μg/mL mitoxantrone for 30 min. Data are presented as the mean ± SD and analyzed by one-way ANOVA (****, *P* < 0.0001). The experiment was carried out in biological triplicate. MTX, mitoxantrone.

On the bacterial genome, a major facilitator superfamily efflux pump gene *smvA* is located next to the *kstR2* gene (Fig. 1B). Given that TetR family proteins usually repress neighboring pump gene expression, we suspected that SmvA might play a role in the increased MTX resistance in the *kstR2* mutants. We then examined our hypothesis using M-3 as the representative strain. The *smvA* gene was upregulated in M-3 (Fig. 2A), and the intracellular accumulation of MTX was decreased by 2.7-fold (Fig. 2B). Deletion of *smvA* in M-3 increased the intracellular accumulation of MTX and reduced the MIC of MTX (Fig. 2B; Table 3). In combination, these results demonstrate that mutations in *kstR2* result in the overexpression of *smvA*, which increases the resistance by reducing the intracellular accumulation of MTX.

### Bacterial response to MTX

**Fig 3:**
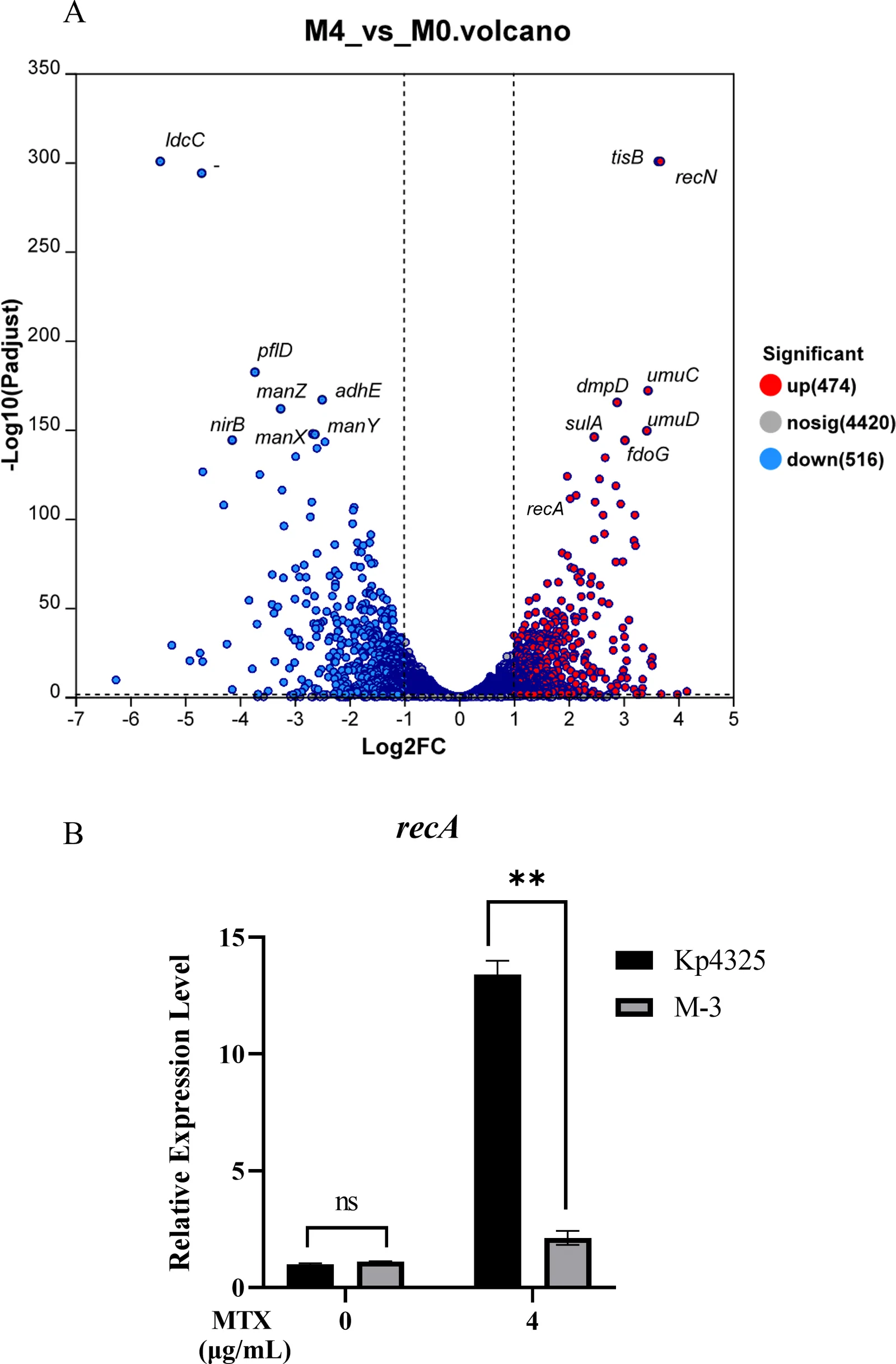
Impact of mitoxantrone on gene expression. (**A**) The volcano plot of differentially expressed genes in Kp4325 after exposure to 4 μg/mL mitoxantrone. The experiment was carried out in biological triplicate. (**B**) mRNA levels of *recA* in Kp4325 and mutant strain M-3 treated by 4 μg/mL mitoxantrone. The *rpoB* gene was used as an internal control. ns, not significant; **, *P* < 0.01 by Student’s *t*-test. The experiment was performed with two technical replicates. Data shown are representative of three independent experiments. MTX, mitoxantrone.

To elucidate the mechanisms underlying the antibacterial activity of MTX, we performed transcriptomic analysis. Treatment with MTX resulted in 474 upregulated and 516 downregulated genes (Fig. 3A). Among the highly upregulated genes, there were SOS response genes, including *recA*, *recN*, *umuC*, and *umuD*. RT-qPCR verified the MTX-induced upregulation of *recA* (Fig. 3B). However, the induction was diminished in strain M-3, presumably due to the reduced intracellular accumulation of MTX (Fig. 3B). Given the role of RecA in bacterial response to DNA damage, we analyzed bacterial genomic DNA following MTX treatment as previously described (26, 27). At the concentrations of 16 and 64 μg/mL, MTX resulted in more DNA smearing compared to the samples treated with lower MTX concentrations, indicating damages of the bacterial genome (Fig. S1).

### MTX synergizes with tigecycline against *K. pneumoniae*

The anti-*K*. *pneumoniae* effect of MTX indicates a potential therapeutic application. To increase treatment efficacy, we utilized a checkerboard assay to identify antibiotics that synergize with MTX against the Kp4325 strain and a carbapenem-resistant strain, Kp43. Among the eight tested antibiotics, tigecycline displayed synergy with MTX against both of the strains (Table 4; Fig. S2).

**TABLE 4:** MICs of antibiotics and FICI values of each antibiotic with MTX^*c*^

Antibiotics	Kp4325^*a*^		Kp43^*a*^	
	MIC (μg/mL)	FICI	MIC (μg/mL)	FICI
TOB^*b*^	0.5	1.5	>512	>1
MEM	0.125	1	>128	>1
TC	8	0.75	256	0.5625
TGC	2	0.375	4	0.25
AZM	0.5	1	32	0.75
ATM/AVI	1	0.75	0.5	0.75
CIP	0.0078	0.53,125	64	0.5
PB	2	1.25	1	1

^*a*^ Data represent results from three independent experiments.

^*b*^ ATM, aztreonam; AVI, avibactam; AZM, azithromycin; CIP, ciprofloxacin; MEM, meropenem; PB, polymyxin B; TC, tetracycline; TGC, tigecycline; TOB, tobramycin.

^*c*^ FICI values under 0.5 indicate a synergistic effect, and values at 0.5 to 1 indicate an additive effect.

**Fig 4:**
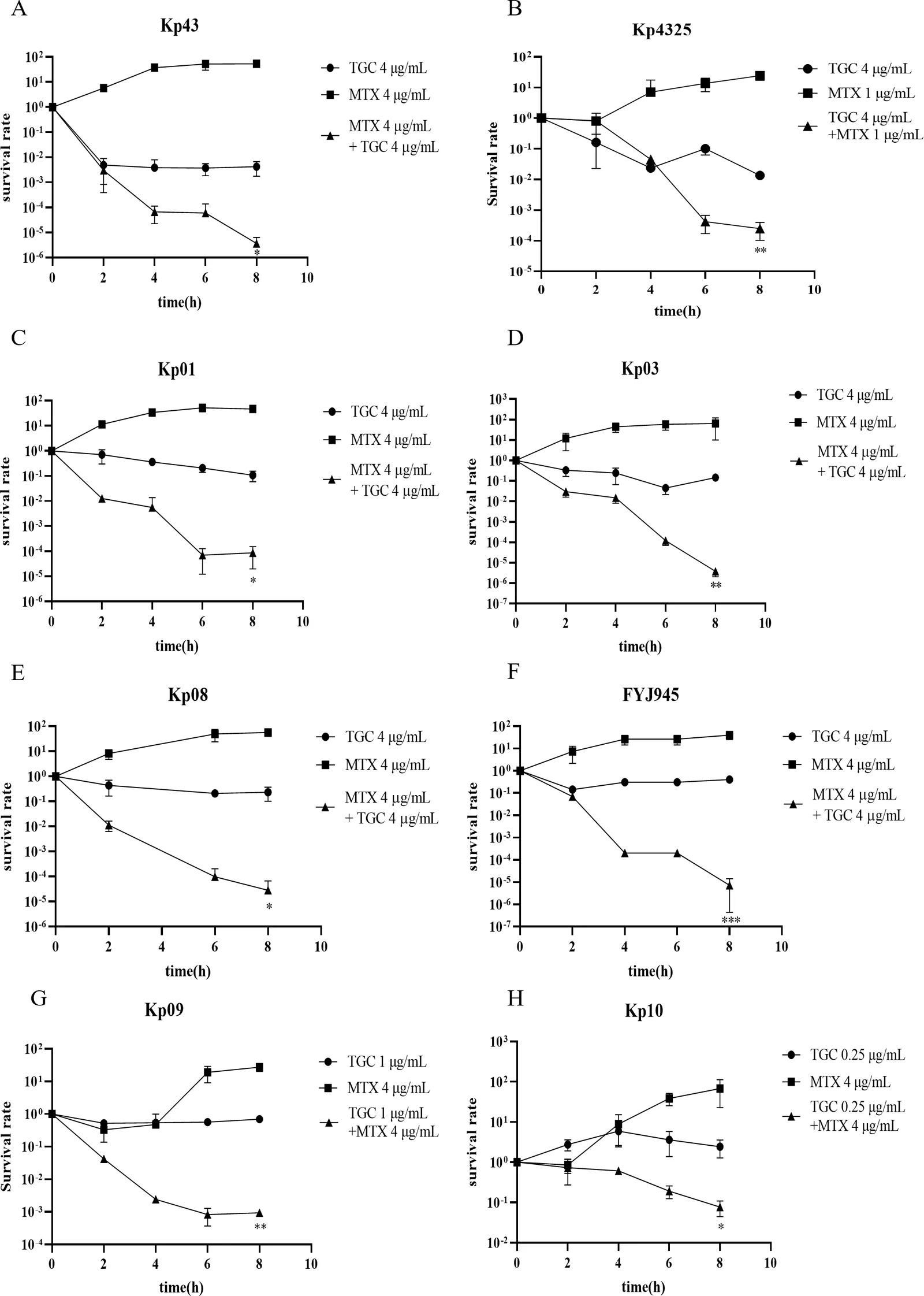
Time-killing curves against *K. pneumoniae* clinical isolates. (**A**) Kp43; (**B**) Kp4325; (**C**) Kp01; (**D**) Kp03; (**E**) Kp08; (**F**) FYJ945 (polymyxin B‑resistant); (**G**) Kp09; (**H**) Kp10. *, *P* < 0.05; **, *P* < 0.01; ***, *P* < 0.001 by Student’s *t*-test. The experiment was carried out in biological triplicate. MTX, mitoxantrone; TGC, tigecycline.

We then assessed the bactericidal effects of MTX in combination with tigecycline. Although MTX alone displayed no bactericidal activity, it increased the killing efficacy of tigecycline against Kp4325 and Kp43 by 108.9 and 1,161.6-fold 8 h after the treatment, respectively (Fig. 4). We further tested the killing efficacies against the clinical isolates (Kp01, Kp03, Kp08, Kp09, Kp10, and FYJ945) (Table S1). Compared to tigecycline alone, MTX increased the killing efficacies by 31.6–5.58 × 10^4^-fold (Fig. 4).

**Fig 5:**
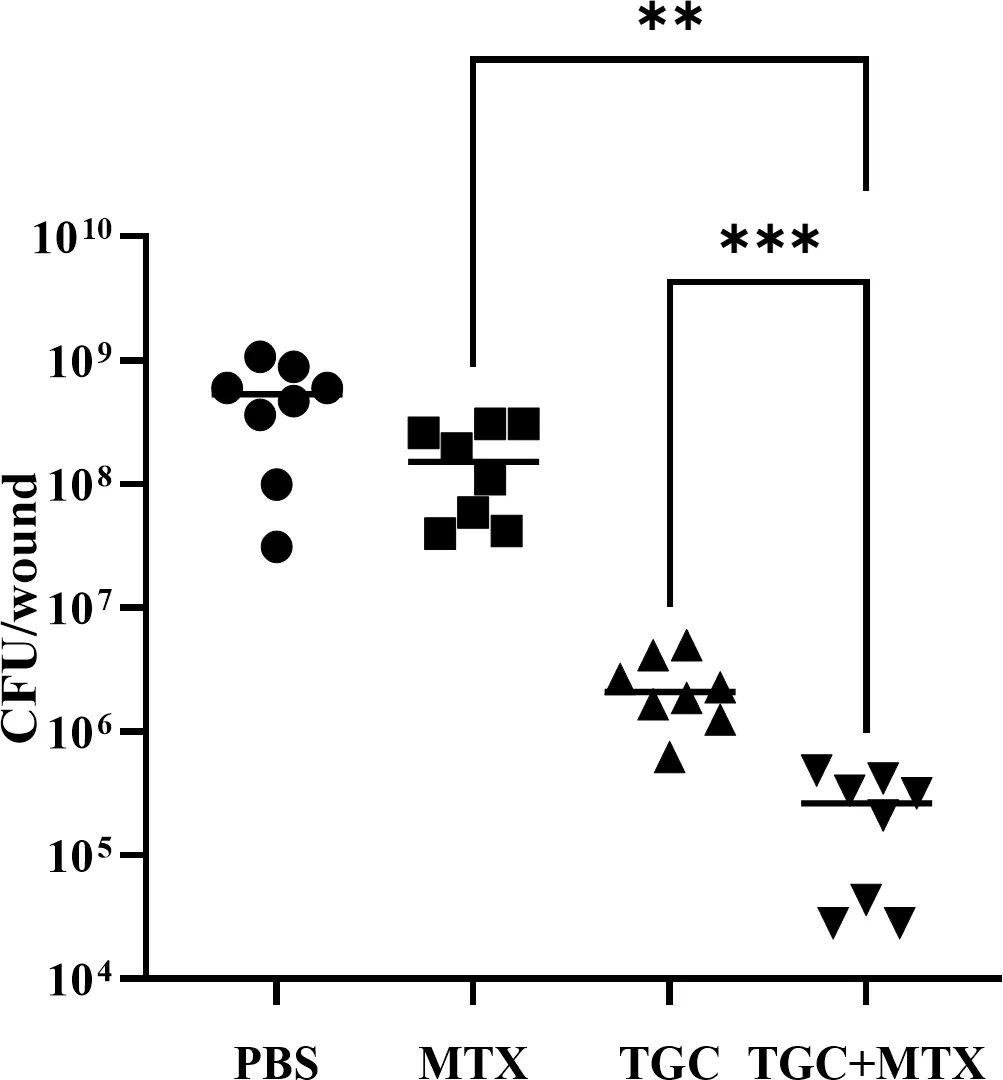
Synergistic effects of tigecycline and mitoxantrone against *K. pneumoniae in vivo*. Bacterial loads of Kp4325 in the wounds of mice 23 h after treatment with mitoxantrone (4 mg/kg) and tigecycline (0.5 mg/kg), alone or in combination (*n* = 8 per group). **, *P* < 0.01;***, *P* < 0.001 by Student’s *t*-test. MTX, mitoxantrone; TGC, tigecycline.

Next, we utilized a mouse wound infection model to examine the *in vivo* bactericidal activities of the combination. In clinical settings, MTX is routinely administered intravenously at 12–14 mg/m² per cycle (approximately 3 weeks) (28). Based on the body surface area cross-species conversion, the equivalent dose for the mouse is 4.65 mg/kg (29). To reduce potential side effects, we utilized 4 mg/kg MTX in the *in vivo* assay, which is within the safe range. Compared to the PBS-treated control group, MTX and tigecycline alone reduced the mean bacterial loads by 2- and 219-fold, respectively (Fig. 5). However, the combination reduced the mean bacterial loads by 1,752-fold (Fig. 5), indicating synergistic bactericidal effects of the combinations *in vivo*.

### Mechanism of the synergy between MTX and tigecycline

**Fig 6:**
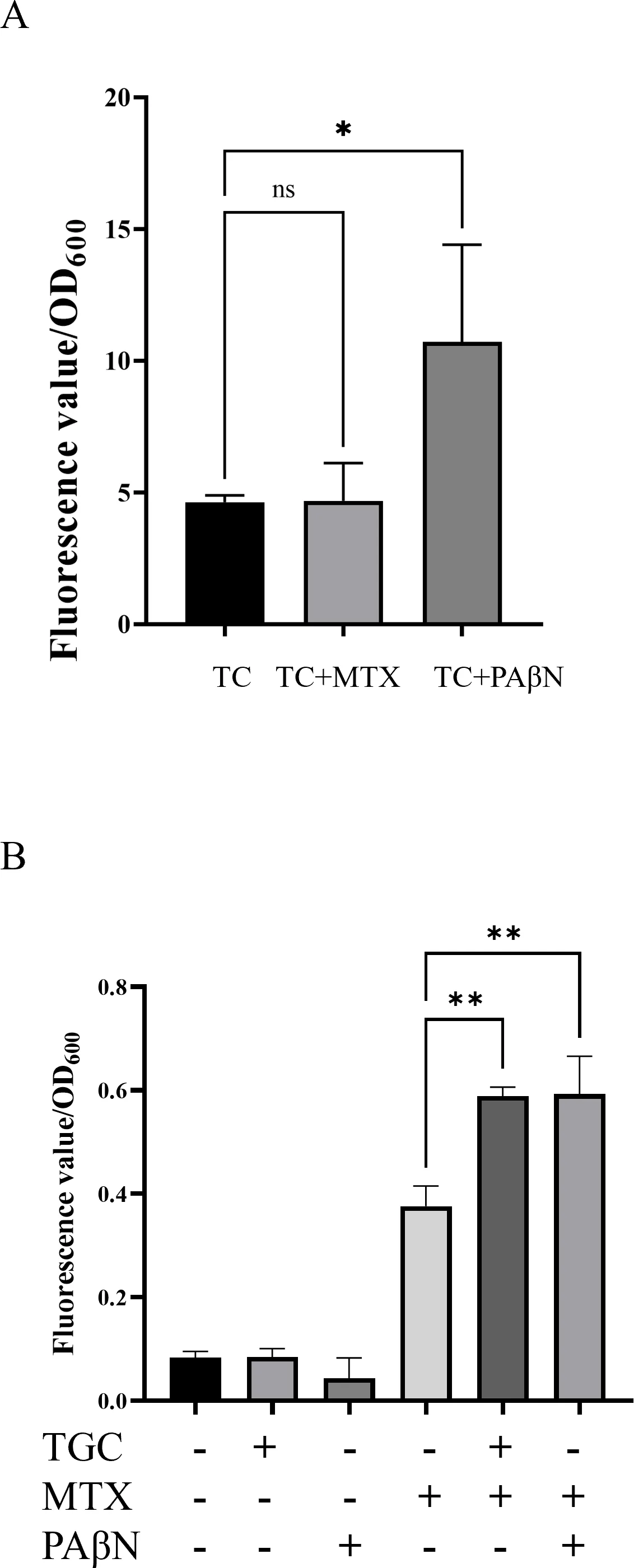
Intracellular accumulation of tetracycline and mitoxantrone in Kp4325. (**A**) Intracellular amounts of tetracycline in Kp4325 after exposure to tetracycline (100 μg/mL) and tetracycline combined with 4 μg/mL mitoxantrone for 30 min. PAβN (50 μg/mL) was added as a positive control. Data are presented as the mean ± SD and analyzed by one-way ANOVA (*, *P* < 0.05). ns, not significant. (**B**) Intracellular accumulation of mitoxantrone in Kp4325 was measured after 30 min of exposure under the following conditions: PBS, tigecycline (0.5 μg/mL), PAβN (50 μg/mL), mitoxantrone (1 μg/mL), tigecycline + mitoxantrone, and PAβ*N* + mitoxantrone. **, *P* < 0.01 by one-way ANOVA. Experiments were carried out in biological triplicate. MTX, mitoxantrone; TC, tetracycline; TGC, tigecycline.

To understand the synergistic mechanism of the MTX-tigecycline combination, we first examined whether the two drugs can promote each other’s influx. Since tigecycline lacks intrinsic fluorescence, we utilized the structure of a similar fluorescent tetracycline to evaluate intracellular accumulation (30). Time-kill curve experiments demonstrated that MTX also synergized with tetracycline against Kp4325 (Fig. S3). The RND-family efflux pump inhibitor PAβN was used as a positive control, which increased tetracycline accumulation in Kp4325 (Fig. 6A). The presence of MTX did not increase intracellular accumulation of tetracycline (Fig. 6A). However, tigecycline increased intracellular accumulation of MTX to a level comparable to PaβN (Fig. 6B).

**Fig 7:**
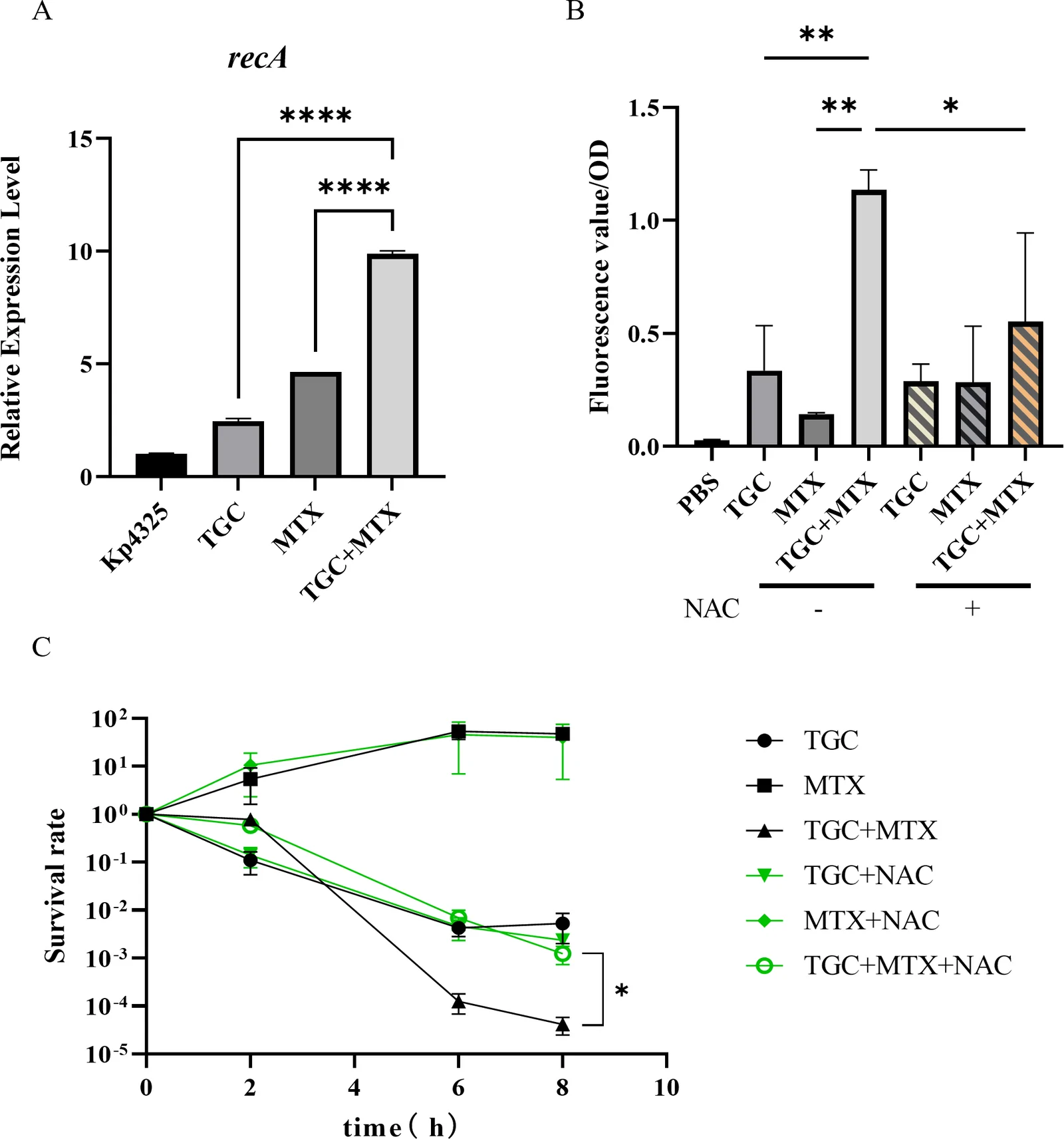
The combination of mitoxantrone and tigecycline enhances SOS response and ROS production in the physiologically relevant medium. (**A**) mRNA levels of *recA* in Kp4325 treated with 0.0625 μg/mL tigecycline and 2 μg/mL mitoxantrone, alone or in combination. The *rpoB* gene was used as an internal control. Data are presented as the mean ± SD and analyzed by one-way ANOVA (****, *P* < 0.0001). The experiment was performed with two technical replicates. Data shown are representative of three independent experiments. (**B**) The production of ROS in Kp4325 after exposure to 0.5 μg/mL tigecycline, 2 μg/mL mitoxantrone, and alone or in combination with or without 5 mM NAC. Data are presented as the mean ± SD and analyzed by one-way ANOVA (*, *P* < 0.05; **, *P* < 0.01). The experiment was carried out in biological triplicate. (**C**) Time-killing of Kp4325 with or without NAC. The bacteria were treated by 4 μg/mL tigecycline, 1 μg/mL mitoxantrone, and the drug combination. *, *P* < 0.05 by Student’s *t*-test. The experiment was carried out in biological triplicate. MTX, mitoxantrone; TGC, tigecycline.

To verify the increased accumulation of MTX, we examined the expression of *recA*. Bacteria were treated with tigecycline, MTX alone, or in combination. After 30 min, the tigecycline-MTX combination resulted in approximately 70% bacterial survival (Fig. S4). Bacterial RNA was collected at this time point. The presence of tigecycline increased the expression level of *recA* compared to MTX alone (Fig. 7A), indicating enhanced bacterial response to DNA damage. NPN and PI staining assays demonstrated that tigecycline did not alter permeability of outer and inner membrane at the concentrations tested, respectively (Fig. S5). These results indicate that tigecycline might enhance MTX accumulation by inducing the expression of uptake proteins or reducing efflux.

It has been demonstrated that antimicrobial-caused damage increases bacterial ROS production, which contributes to the bactericidal effect (31). Thus, using the 2′,7′-dichlorodihydrofluorescein diacetate (DCFH-DA) probe, we assessed the intracellular ROS levels in Kp4325 treated with MTX, tigecycline alone, or in combination. Compared to each of the drugs alone, the combination boosted the generation of ROS (Fig. 7B). Addition of the ROS scavenger N-acetyl-L-cysteine reduced the intracellular ROS level and bactericidal efficacy of the MTX-tigecycline combination (Fig. 7B and C). Collectively, these findings demonstrate that the combination of MTX and tigecycline increases MTX intracellular accumulation, which enhanced bacterial ROS production and bactericidal effect.

## DISCUSSION

Drug repurposing provides a cost-effective therapeutic solution against antibiotic-resistant bacteria. Here in this study, we found that MTX is effective against *K. pneumoniae* in the physiologically relevant medium. MTX is an anthracenedione with proven anticancer activity. It was approved by the FDA in 1987 for the treatment of acute myeloid leukemia and is now licensed worldwide for multiple malignancies (32). Compared with structurally related anthracyclines (e.g., doxorubicin and daunorubicin), it displays markedly lower cardiotoxicity and cellular toxicity (33). Its antitumor mechanisms include (i) intercalation into double-stranded DNA (34) and (ii) poisoning of eukaryotic topoisomerase II by trapping the cleavage complex, thereby blocking religation and generating lethal DNA double-strand breaks (35). Bacterial DNA gyrase is functionally and mechanistically similar to eukaryotic topoisomerase II. We therefore hypothesized that MTX might induce DNA damage by inhibiting bacterial DNA gyrase and intercalating into DNA. Supporting this, our RNA-seq data revealed upregulation of SOS response genes after MTX exposure, presumably due to DNA damage. By passaging a wild-type *K. pneumoniae* strain under MTX pressure in the physiologically relevant medium, we obtained resistant mutants whose MTX MICs rose ≥1,024-fold. Whole-genome sequencing of three independent mutants revealed mutations within the N-terminal DNA-binding domain of the TetR-family transcriptional regulator gene *kstR2*. The TetR family transcriptional regulators (TFRs) constitutes a large, ubiquitous class of one-component transcriptional regulators (36). Each member possesses an N-terminal helix-turn-helix DNA-binding domain and a C-terminal ligand-sensing domain (37). TFR usually acts as a transcriptional repressor to regulate the expression of the adjacent efflux pump (38). In this study, mutations in *kstR2* upregulated *smvA* expression. This upregulation reduced intracellular MTX accumulation, ultimately increasing the MICs of the *kstR2* mutants to 256 or 512 μg/mL. In addition to *kstR2*, point mutations in the *fur* gene were also identified in all three resistant mutants. Fur is an iron-responsive global transcriptional regulator (39). It directly represses at least six iron uptake systems in *K. pneumoniae*, including the synthesis and uptake genes for siderophores, such as enterobactin, aerobactin, salmochelin, and yersiniabactin (40). Furthermore, Fur indirectly controls capsular polysaccharide (CPS) synthesis by repressing the transcription of *rmpA* (41). The role of Fur in antibiotic resistance remains largely unknown. Mutation in the *fur* gene has been shown to affect the production of CPS (42), and hyperproduction of CPS in *K. pneumoniae* increases bacterial susceptibility to polymyxins (43). Further studies are warranted to investigate the influence of the *fur* mutation on MTX resistance.

We found that MTX and tigecycline act synergistically against *K. pneumoniae*. Tigecycline increased intracellular MTX accumulation, which resulted in enhanced expression of SOS response genes and ROS production, likely due to increased DNA damage. We suspected that tigecycline might increase membrane permeability or reduce efflux of MTX. Further studies, such as transcriptomic and proteomic analyzes are warranted to elucidate the mechanism.

MTX was previously shown to reprogram macrophages (44). The drug recruits macrophages, potentiates their microbicidal activity, and upregulates pro-inflammatory cytokines, triggering a protective immune response that ultimately eradicates the pathogen *in vivo* (45). A previous study on *Salmonella enterica* and *Escherichia coli* revealed that MTX retains full activity regardless of the presence of high-profile resistance determinants, such as *bla_NDM_*, *mcr*, or *tet(X4*), which normally render bacteria resistant to last-resort antibiotics (18). Despite these promising properties, as an anthracenedione anticancer agent, MTX carries toxic liabilities (e.g., cardiotoxicity and myelosuppression) (46, 47), which necessitate careful consideration regarding its clinical repurposing. In the murine wound infection model, MTX was administered once at 4 mg/kg, which is lower than the equivalent human dose in clinical settings (28, 29). Considering that antibacterial therapy typically requires a shorter treatment duration compared to prolonged or cyclic oncological regimens, the risk of cumulative adverse effects might be alleviated.

To improve the therapeutic index, targeted delivery and structural optimization are being pursued. Encapsulation in liposomes markedly reduces cardiotoxicity while retaining antineoplastic potency (48). Likewise, embedding MTX in chitosan-coated PLGA nanospheres provides sustained, controlled release, minimizing peak-related adverse effects and enhancing local drug exposure (49). These nanocarrier strategies offer a potential therapy toward potent antibacterial therapy with an acceptable safety profile. Conjugation with bacteria-targeting antibodies is another strategy to reduce side effects and enhance treatment efficacy.

In summary, we identified the non-antibiotic drug MTX as a potent anti-*K*. *pneumoniae* agent that induces bacterial DNA damage. We further demonstrated synergy between tigecycline and MTX, which provides a potential treatment strategy against multidrug-resistant *K. pneumoniae*.

## MATERIALS AND METHODS

### Bacterial strains, plasmids, primers, and growth conditions

The bacterial strains, plasmids, and primers used in the study are listed in Table S4. The *K. pneumoniae* strains were cultured in LB or the physiologically relevant medium (RPMI with 5% MHB) at 37°C (23). Gene deletion and complementation were performed as previously described (50). Briefly, the spacer targeting *smvA* was designed using sgRNAcas9 software and cloned into pSGKP-km to generate pSGKP-*smvA*. Homologous arms were PCR-amplified from genomic DNA of strain M-3 and fused to form a linear fragment by overlap PCR. The pCasKP-apr plasmid, pSGKP-*smvA*, and the linear homologous fragment were co-electroporated into M-3 bacteria cells. Transformants were selected on LB agar containing apramycin (30 μg/mL) and kanamycin (50 μg/mL). Colonies were screened by colony PCR. Positive clones were cured of both plasmids by growth at 37°C and selected on an LB plate containing 5% sucrose, resulting in the unmarked *smvA* knockout strain.

### Antimicrobial susceptibility tests

The MICs were determined using the physiologically relevant medium by the twofold serial dilution method (51). Bacteria were cultured in LB or the physiologically relevant medium to an OD_600_ of 1 and diluted 1,000-fold in the physiologically relevant medium, resulting in a concentration of 5 × 10^5^ CFU/mL. The bacterial suspension (200 μL) was inoculated into each well of a 96-well plate, followed by incubation at 37°C for 24 h without shaking. The MICs were examined in triplicate.

### *In vitro* evolution of MTX-resistant strains

Kp4325 was passaged in the presence and absence of MTX. An overnight culture of the bacteria was subcultured into fresh physiologically relevant medium with increasing concentrations of MTX (0.25×, 0.5×, and 1× MIC). After 24 h, cells with the highest concentration of MTX that reached an OD_600_ of ≥2.0 were inoculated into fresh physiologically relevant medium with increased concentrations of MTX (such as 0.5×, 1×, and 2× MIC) for another round of passage. The passaging was repeated for 13 days, and the replicates were streaked to obtain single colonies for MIC determination.

### Genome sequencing

Genomes of the MTX-resistant mutant colonies M-1, M-2, and M-3 and two control strains passaged in the physiologically relevant medium (designated C-1 and C-2) were sequenced by the Illumina HiSeq X Ten platform after quality control. Both sequencing and subsequent analysis were conducted by the Tianjin Uniteomics Company. Paired-end raw reads were filtered using the fastp preprocessor. To identify acquired mutations, the clean reads of the MTX-resistant mutants were mapped directly to the sequencing assembly of the control strain, which served as the reference template, using BWA software. *GATK*, snpEff, and vcftools were subsequently utilized for variant calling and annotation of single-nucleotide polymorphisms and small insertions/deletions (indels).

### RT-qPCR

Overnight bacteria were cultured to an OD_600_ of 0.5. The bacteria were then treated with indicated drugs in the physiologically relevant medium for 30 min. After the treatment, bacterial survival was first determined by dilution spotting, followed by RNA extraction. The extracted RNA (1 μg) was subjected to reverse transcription with the HiScript III RT SuperMIX for qPCR (+gDNA wiper) (Vazyme, China) following the manufacturer’s protocol. RT-qPCR was performed by PerfectStart Green qPCR SuperMix (TransGene Biotech, China).

### Transcriptomic analysis

The overnight bacteria were cultured to an OD_600_ of 0.5. Kp4325 was cultured in the physiologically relevant medium, then treated with or without MTX (4 μg/mL) for 30 min followed by RNA extraction. RNA sequencing was performed by Majorbio-Shanghai following the standard operational procedure. Data analysis was performed using the Majorbio Cloud (www.majorbio.com).

### DNA fragmentation assay

To evaluate DNA fragmentation in bacteria treated with MTX, genomic DNA extraction and agarose gel electrophoresis were performed (26, 27). Briefly, 2 mL Kp4325 (2.5 × 10^8^ CFU/mL) treated with MTX (4, 16, 64 μg/mL) was harvested by centrifugation, lysed, and incubated with proteinase K to remove proteins, followed by RNase A digestion. DNA was then purified by chloroform-phenol extraction, precipitated with isopropanol, and resuspended in ddH_2_O. Equal amounts of DNA were resolved on 1% agarose gel and stained with StarStain Red Plus Nucleic (GenStar, Beijing, China) to visualize fragmentation.

### Checkerboard assay

The synergistic effect between antibiotics was examined by using the chessboard experiment method. Briefly, indicated antibiotics and MTX were diluted with the physiologically relevant medium into 96-well plates in either horizontal or vertical coordinates, respectively. After incubation at 37°C for 20 h, the bacterial growth was assessed by measuring OD_600_. The MIC values were defined as the lowest concentrations of antibiotics with no visible growth of bacteria. The FICI was calculated according to the previously described formula: FICI = FIC_a_ + FIC_b_ = MIC_ab_ / MIC_a_ + MIC_ba_ / MIC_b_ (52). Synergy was defined as an FIC index of ≤0.5.

### Time-dependent killing assays

Bacteria were cultured to an OD_600_ of 1.0 in LB and then diluted 10-fold into fresh physiologically relevant medium, resulting in 5 × 10^7^ CFU/mL. The cells were treated with either tigecycline and MTX alone or their combination at 37°C with agitation at 200 rpm. At indicated time points (0, 2, 4, 6, and 8 h), viable bacterial counts were determined by plating on LB agar. All experiments were conducted in triplicate.

### Mouse wound infection model

The procedure for mouse wound infections was modified from a previous study (53). Kp4325 was cultured in LB until the OD_600_ reached 1.0. The bacteria were washed twice with PBS and resuspended to a concentration of 2 × 10^8^ CFU/mL. The fur on the back of each female BALB/c mouse (8 weeks old, weighing approximately 20 g) was removed by shaving, followed by the application of chemical depilatories. The skin was then disinfected with 70% ethanol before creating a 6 mm full-thickness wound. The bacteria (20 μL) were added to the wound, resulting in 4 × 10^6^ CFU per mouse. MTX (20 μL), tigecycline, the MTX and tigecycline combination, or PBS was added to the wound area at 1 h postinfection. Twenty-four hours after the infection, the mice were euthanized, and 1 cm × 1 cm square skin around the wound was removed and collected in sterile 1% proteose peptone (Solarbio, Beijing, China). The skin samples were homogenized. The bacterial burdens were determined through serial dilution with saline and plating.

### Tetracycline accumulation assays

Tetracycline accumulation was determined as previously described with minor modifications (54). Kp4325 was cultured in LB until the OD_600_ was 1.0. One milliliter of the bacteria was collected by centrifugation and resuspended in 1 mL physiologically relevant medium, followed by incubation with 100 mg/L tetracycline or in combination with 4 mg/L MTX for 15 min. The bacteria were washed with 1 mL of Mg^2+^ buffer (50% methanol, 10 mM Tris-HCl, 0.1 mM MgCl_2_, 0.2% glucose, pH 8.0), resuspended in 1 mL of the Mg^2+^ buffer. The bacterial suspension (200 μL) was added to each well of a 96-well plate, followed by measurement of the fluorescence (excitation = 400 nm, emission = 520 nm) using the Varioskan Flash reader (Thermo Scientific, Netherlands). The efflux pump inhibitor PAβN (50 mg/L) was incubated with tetracycline as a positive control.

### MTX uptake assay

Overnight bacteria were subcultured at 37°C until the cell density reached OD_600_ = 0.5. The cells were treated with MTX alone or in combination with tigecycline in the physiologically relevant medium. After 30 min of incubation at 37°C, 1 mL aliquot was removed from each sample and washed three times with PBS. Then, 200 μL of each sample was transferred to a well of a black-wall 96-well plate, and the MTX fluorescence (excitation = 610 nm, emission = 685 nm) was measured (55). MTX levels were normalized to bacterial OD_600_.

### Assessment of outer and inner membrane permeability

Outer and inner membrane permeability were assessed by the NPN uptake assay and propidium iodide (PI) staining, respectively, as previously reported with minor modifications (56, 57). Overnight bacterial cultures were diluted 1:50 in LB and grown to an OD_600_ of 1 at 37°C. The cells were then adjusted to OD_600_ = 0.5 in fresh physiologically relevant medium and incubated with or without tigecycline (0.25-0.5 μg/mL) for 0.5 h at 37°C. Then the cells were washed three times with 5 mM HEPES buffer (pH 7.2) containing 5 mM glucose (for outer membrane assay) or with PBS (for inner membrane assay). For measuring outer membrane permeability, NPN (1-N-phenylnaphthylamine) was added to a final concentration of 10 μM, and fluorescence was measured at excitation at 350 nm and emission at 420 nm. For measuring inner membrane permeability, PI was added to 10 μM, incubated at 25°C for 30 min, and fluorescence measured at excitation at 535 nm and emission at 615 nm. All assays were performed in triplicate.

### ROS measurement

The amount of ROS was determined as previously described with minor modifications (58). Cells were treated by either tigecycline or MTX alone or in combination in the physiologically relevant medium for 30 min in the presence of the ROS probes DCFH-DA at a concentration of 30 μM. Cells were washed three times with PBS and resuspended in PBS, followed by measurement of the fluorescence (excitation = 488 nm, emission = 525 nm).

## Data Availability

The WGS data of the evolved Kp4325 mutants were deposited in the NCBI database under BioProject accession number PRJNA1393886. The raw RNA-seq reads have been deposited in the NCBI Sequence Read Archive under BioProject accession number PRJNA1393935.

## SUPPLEMENTAL MATERIAL

Fig. S1 to S5 spectrum.00568-26-s0001.pdf DNA fragmentation assay, checkerboard assay, time-kill curves, bacterial viability, and membrane permeability.

Supplemental tables spectrum.00568-26-s0002.pdf MICs (mg/L) of indicated *K. pneumoniae* strains; ST and KL types of indicated *K. pneumoniae* strains; mutations in the mitoxantrone-resistant strains; bacteria, plasmids, and primers used in this study.

ASM does not own the copyrights to Supplemental Material that may be linked to, or accessed through, an article. The authors have granted ASM a non-exclusive, world-wide license to publish the Supplemental Material files. Please contact the corresponding author directly for reuse.
