# Supplementary material for: Mitoxantrone synergies with tigecycline against Klebsiella pneumoniae
Source: Microbiol Spectr. 2026 Aug 12;14(9):e00568-26. doi: 10.1128/spectrum.00568-26 (PMC13532325; doi:10.1128/spectrum.00568-26)
Supplement: Supplementary file 1 [file spectrum.00568-26-s0001.pdf]

Figure. S1

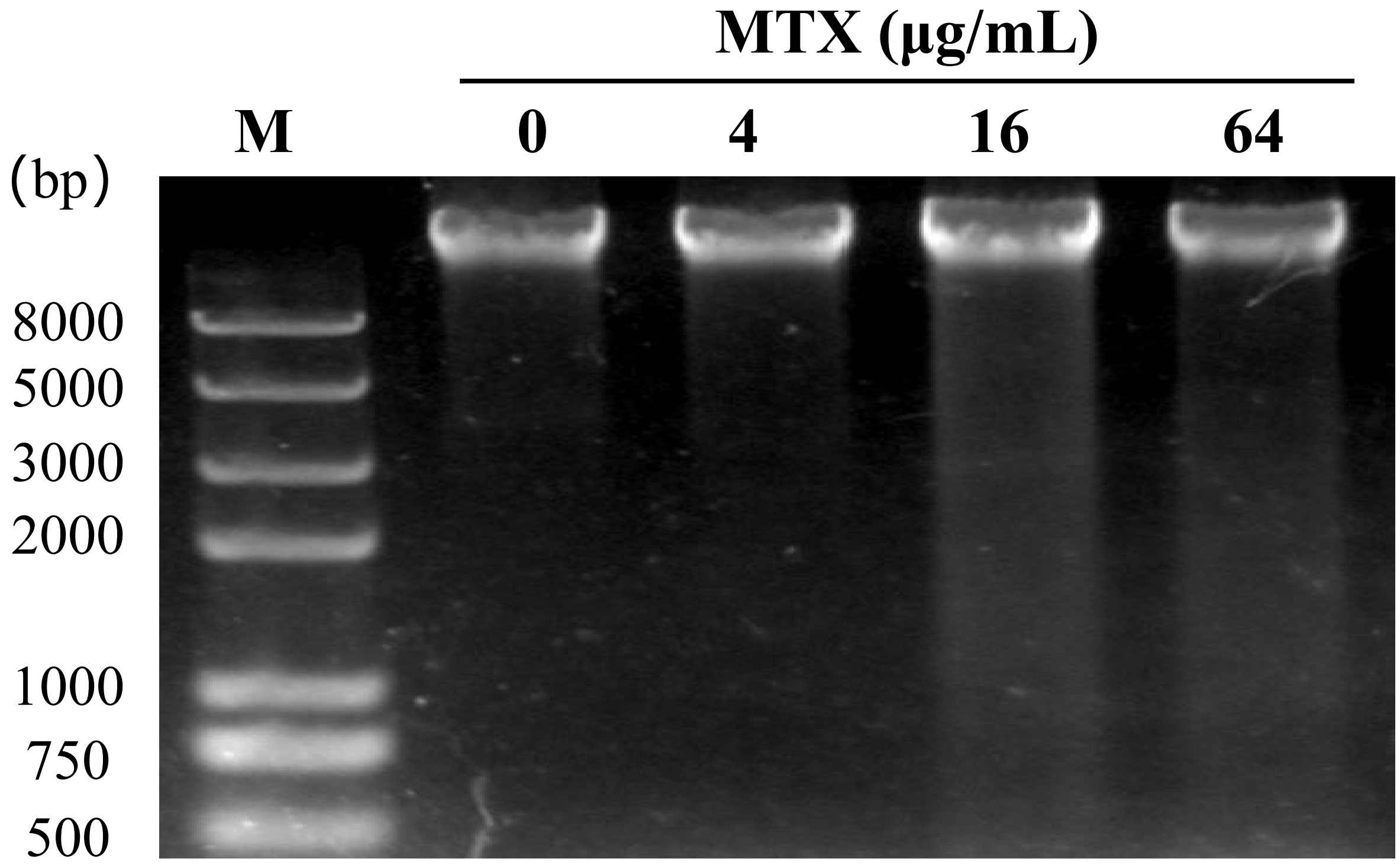

Figure. S1. DNA fragmentation assay for mitoxantrone. treated Kp4325. Lanes respectively represent the untreated control, the treated Kp4325 with 4, 16, 64 μg/mL MTX. M: Marker; MTX: mitoxantrone.

Figure. S2

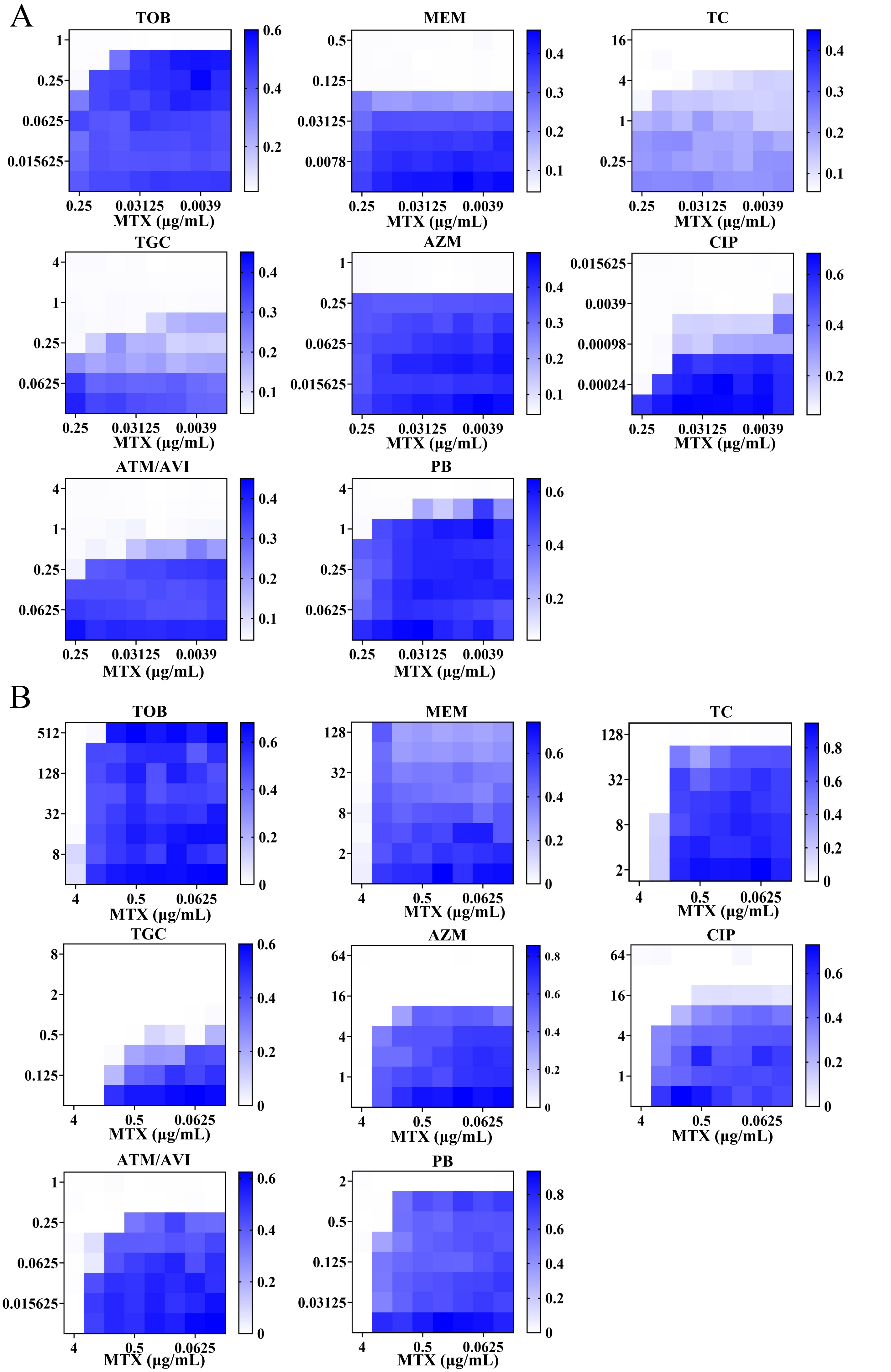

Figure. S2. Checkerboard assay of mitoxantrone and different antibiotics against Kp4325 and Kp43. Heatmaps showing the in vitro combined antibacterial effects of mitoxantrone with different antibiotics against KP4325 (A) and KP43 (B). MTX: mitoxantrone; TOB: Tobramycin; MEM: Meropenem; TC: Tetracycline; TGC: Tigecycline; AZM: Azithromycin; ATM: Aztreonam; AVI: Avibactam; CIP: Ciprofloxacin; PB: Polymyxin B

Figure. S3

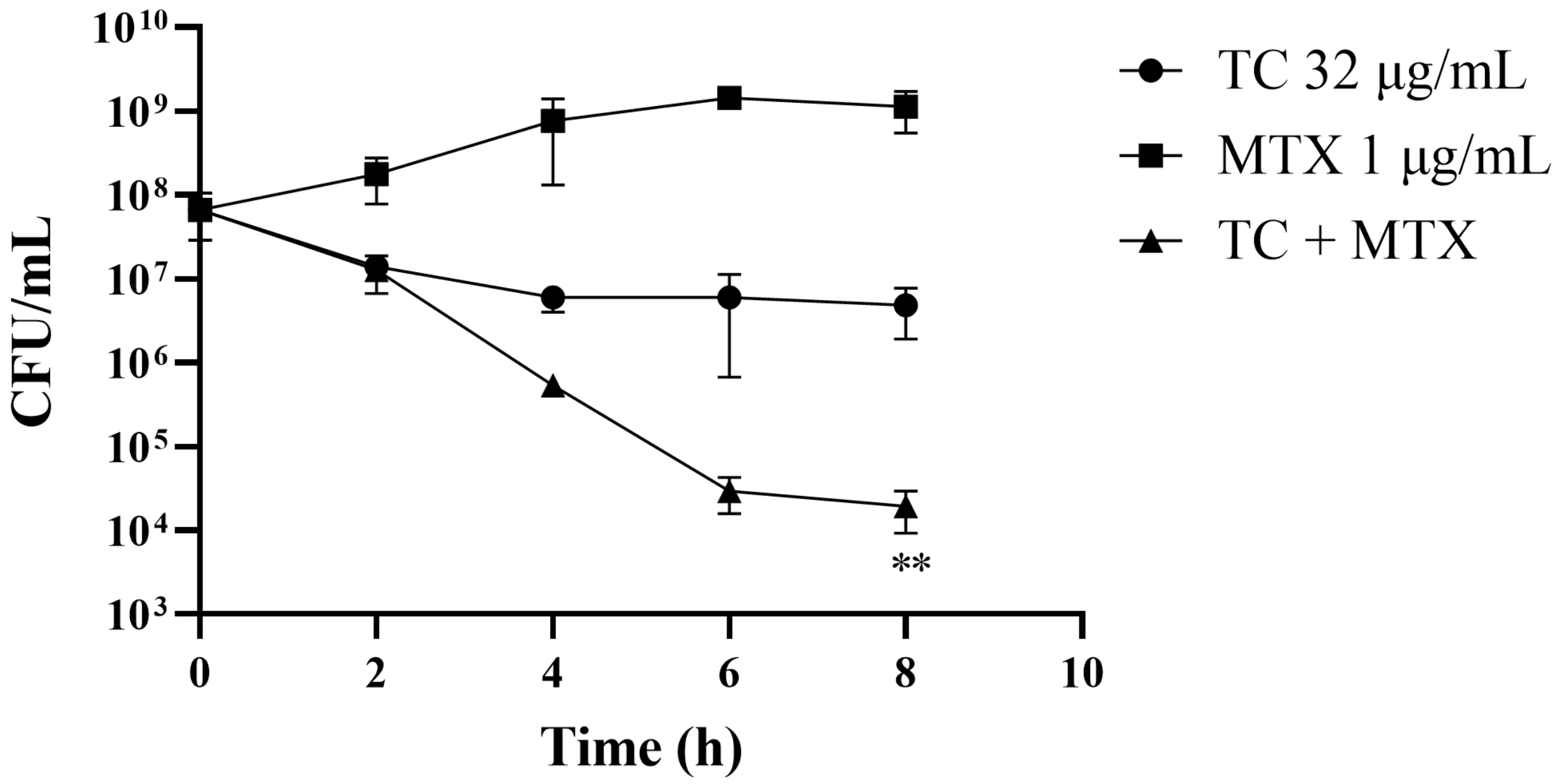

Figure. S3. Time-kill curves of TC and MTX against KP4325. Data are mean ± SD (n = 3). The combination shows synergistic killing at 8 h. Data are representative of three independent experiments. **, *P* <0.01 by Student's t-test. TC: Tetracycline; MTX: mitoxantrone.

Figure. S4

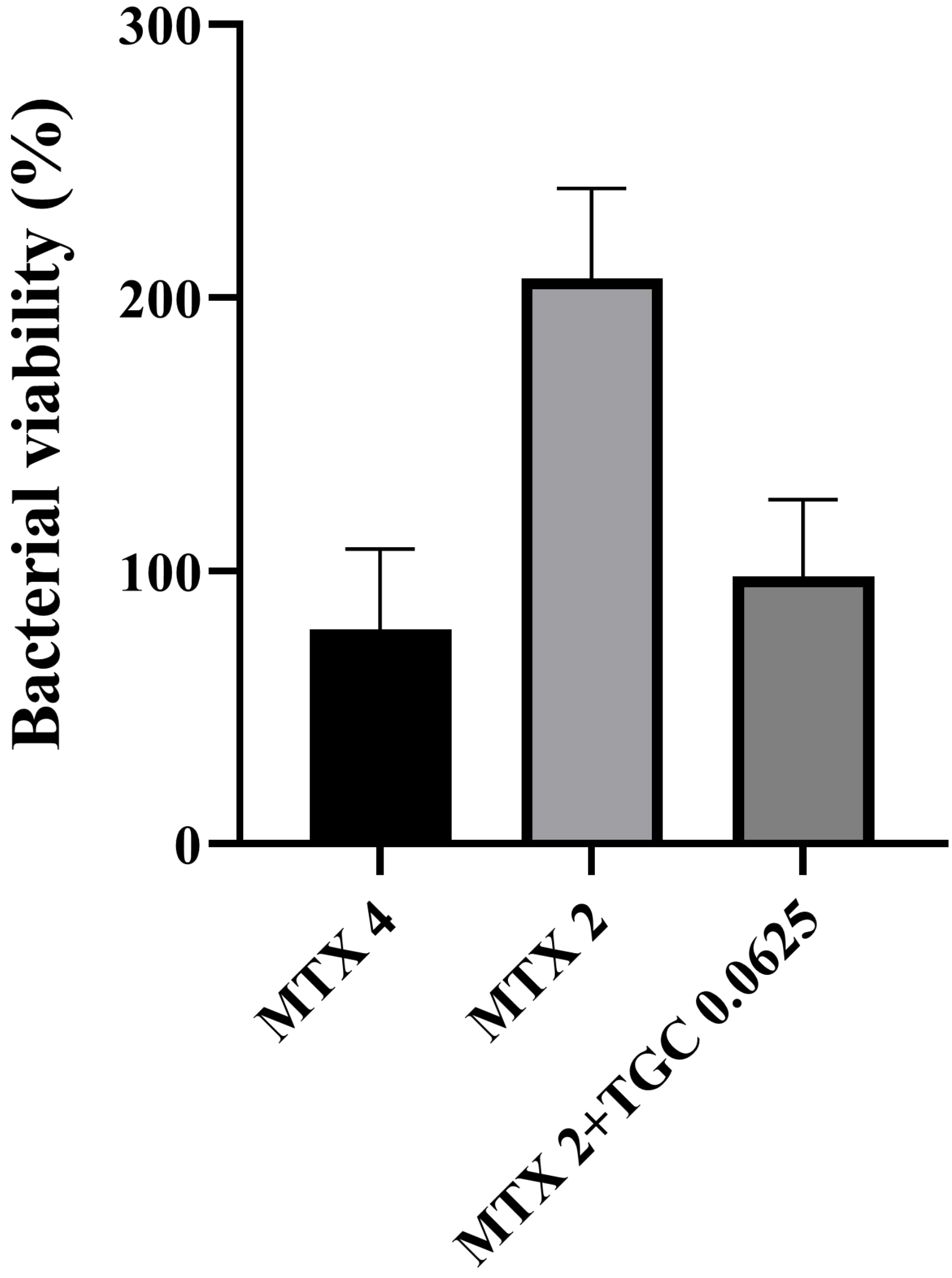

Figure. S4. Bacterial viability after treatment. Bacterial survival was measured after 30 min of exposure to MTX 4 μg/mL, 2 μg/mL and 2 μg/mL MTX combined with 0.0625 μg/mL TGC. The viability remained above 70% (mean ± SD), indicating that the treatment condition preserves sufficient live cells for subsequent analyses. Data are representative of three independent experiments. MTX: mitoxantrone; TGC: Tigecycline.

Figure. S5

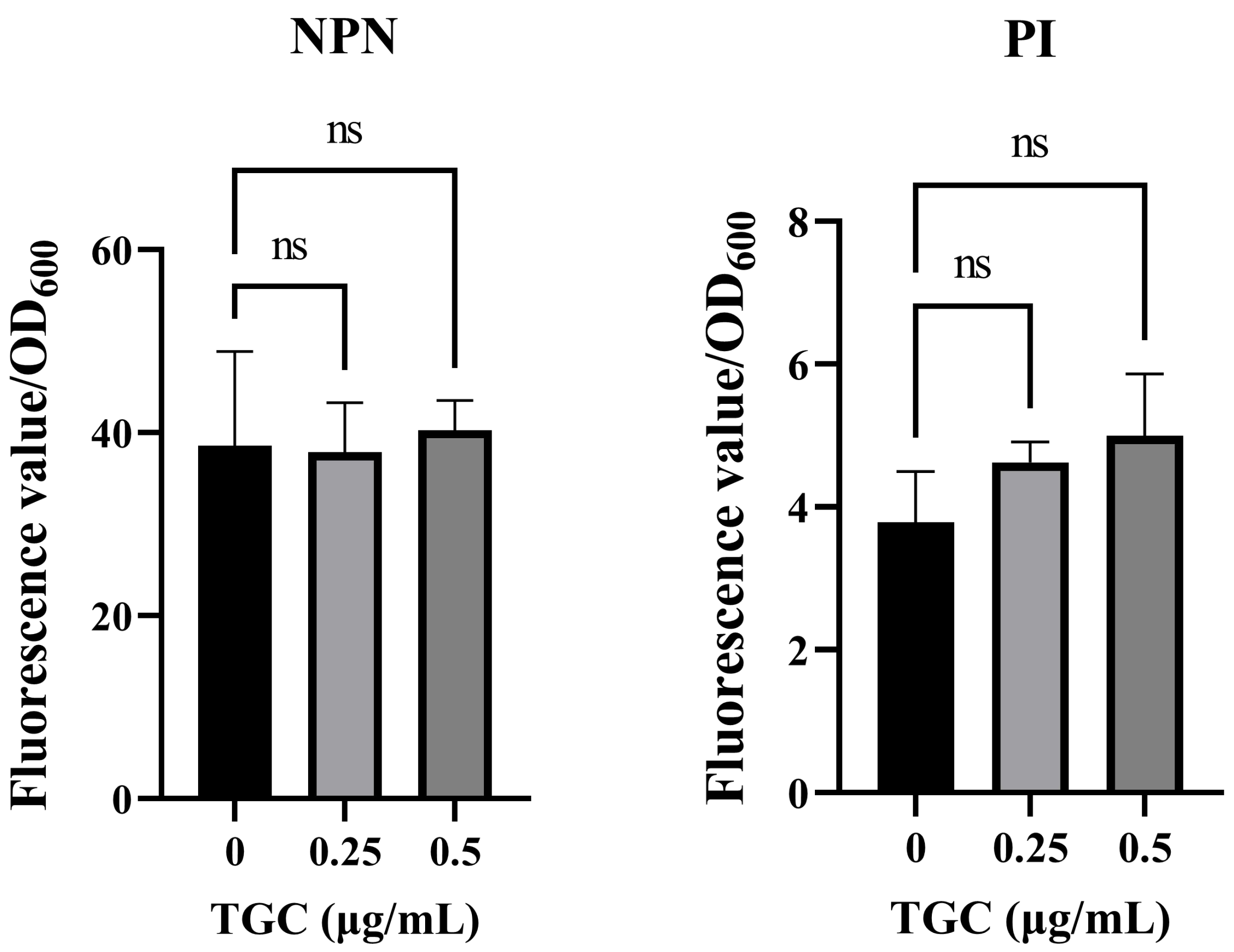

Figure. S5. Effect of TGC on outer membrane permeability. NPN (1-N-phenylnaphthylamine) and PI (propidium iodide) assays were performed to assess outer membrane integrity. No significant change in fluorescence was observed following TGC treatment compared to the control, indicating that TGC does not disrupt outer membrane permeability. Data are representative of three independent experiments (mean ± SD) and analyzed by one-way ANOVA. ns, not significant. TGC: Tigecycline
