# Supplementary material for: Mitoxantrone synergies with tigecycline against Klebsiella pneumoniae
Source: Microbiol Spectr. 2026 Aug 12;14(9):e00568-26. doi: 10.1128/spectrum.00568-26 (PMC13532325; doi:10.1128/spectrum.00568-26)
Supplement: Supplementary file 2 [file spectrum.00568-26-s0002.pdf]

**Table S1.** MICs (mg/L) of indicated *K. pneumoniae* strains.

| | strain | MIC |
|---|---|---|
| MEM | Kp01 | 8 |
| | Kp03 | >16 |
| | Kp08 | 16 |
| | Kp09 | >16 |
| | Kp10 | >16 |
| | Kp43 | >16 |
| PB | FYJ945 | 4 |

MEM, Meropenem; PB: Polymyxin B.

**Table S2. ST and KL type of clinical isolates.**

| strain | ST type | KL type |
|---|---|---|
| Kp01 | 11 | 16/143 |
| Kp03 | 11 | 16/143 |
| Kp08 | 1883 | 16/143 |
| Kp09 | 11 | 47 |
| Kp10 | 11 | 16/143 |
| Kp43 | 11 | 47 |

**Table S3.** Mutations in the mitoxantrone-resistant strains.

| Strains | Mutation | Gene | Product |
|---|---|---|---|
| M-1 | c.A865G p.Ile289Val | Uncharacterised gene | Uncharacterised protein |
| | c.G271A p.Ala91Thr | *fur* | Fur family transcriptional repressor |
| | c.T1355C c.C1679T p.Val452Ala p.Ala560Val | Uncharacterised gene | Uncharacterised protein |
| | c.262_263insCCGCCTG p.Trp88fs | *kstR2* | TetR family transcriptional regulator |
| M-2 | c.A865G p.Ile289Val | Uncharacterised gene | Uncharacterised protein |
| | c.G271A p.Ala91Thr | *fur* | Fur family transcriptional repressor |
| | c.T1355C c.C1679T p.Val452Ala p.Ala560Val | Uncharacterised gene | Uncharacterised protein |
| | c.16_48del33 p.Arg6_Met16del | *kstR2* | TetR family transcriptional regulator |
| M-3 | c.A865G p.Ile289Val | Uncharacterised gene | Uncharacterised protein |
| | c.T206C c.G271A p.Val69Ala p.Ala91Thr | *fur* | Fur family transcriptional repressor |
| | c.T1355C c.T1364C c.C1559T | Uncharacterised | Uncharacterised protein |

| | | |
|---|---|---|
| c.C1679T<br>p.Val452Ala p.Ile454Thr<br>p.Pro519Leu p.Ala560Val | gene | |
| c.167del17<br>p.Asp56fs | *kstR2* | TetR family transcriptional regulator |

**Table S4.** Bacteria, plasmids, primers used in this study.

| | Description | | Source |
|---|---|---|---|
| Bacteria | | | |
| Kp4325 | *K. pneumoniae* clinical isolates | | Lab stock |
| Kp01 | *K. pneumoniae* clinical isolates | | Lab stock |
| Kp03 | *K. pneumoniae* clinical isolates | | Lab stock |
| Kp08 | *K. pneumoniae* clinical isolates | | Lab stock |
| Kp09 | *K. pneumoniae* clinical isolates | | Lab stock |
| Kp10 | *K. pneumoniae* clinical isolates | | Lab stock |
| Kp43 | *K. pneumoniae* clinical isolates | | Lab stock |
| FYJ945 | *K. pneumoniae* clinical isolates | | Lab stock |
| M-1 | Mitoxantrone-resistant *K. pneumoniae* evolved from parental strain Kp4325 | | This study |
| M-2 | Mitoxantrone-resistant *K. pneumoniae* evolved from parental strain Kp4325 | | This study |
| M-3 | Mitoxantrone-resistant *K. pneumoniae* evolved from parental strain Kp4325 | | This study |
| C-1 | Mitoxantrone-sensitive *K. pneumoniae* evolved from parental strain Kp4325 | | This study |
| C-2 | Mitoxantrone-sensitive *K. pneumoniae* evolved from parental strain Kp4325 | | This study |
| M-3Δ*smvA* | M-3 deletion of *smvA* | | This study |
| plasmid | | | |
| pCasKP-apr | Gene expression vector in *K. pneumoniae*, $Apr^r$ | | |
| pSGKP-km | Plasmid express sgRNA in *K. pneumoniae*, $Km^r$ | | |
| pSGKP_*smvA* | pSGKP-km derivative with *smvA* spacer, $Km^r$ | | This study |
| pUCP24 | Shuttle vector between *E. Coli* and *P. aeruginosa* pUCP24 | | This study |
| pUCP24-*kstR2* | *kstR2* gene from Kp4325 cloned in pUCP24, $GM^r$ | | This study |
| primer | Sequence 5'-3' | Function | Source |
| *kstR2*-F | CTTTTTGTTACACTCGGCCAAA | Cloning of the *kstR2* gene in pUCP24 | This study |
| *kstR2*-R | CTAAAACAACTCCAGCGTAAT | | |
| *smvA*-up-F | ACATATTGCCCTCTACTTGC | For deletion of *smvA* | This study |
| *smvA*-up-R | CAGCAGCAGAAGCAATACACGTGACATAGCTTGTACCTC | | |

| | | | |
|---|---|---|---|
| *smvA*-dn-F | GAGGTACAAGCTATGTCACGTGTATTGCTTCT GCTGCTG | | |
| *smvA*-dn-R | TTGCGCAAGGTATAGTGATC | | |
| *smvA*-spacer-F | TAGTGCGTGAGGCAATCAGCGTCA | | |
| *smvA*-spacer-R | AAACATATCCGCCTGTGGCGCGAA | | |
| *recA*-qPCR-F | GATGAGCCAGGCGATGCGTAA | qPCR | This study |
| *recA*-qPCR-R | CGATGCGGCGAATGTCCAGA | | |
| *smvA*-qPCR-F | TGATGGCGATTTTGCTGGTTT | | |
| *smvA*-qPCR-R | CTCATTGCCGCTGCTACCCA | | |
| *rpoB*-qPCR-F | TCAGTTCGCCAGTCGCTTCATC | | |
| *rpoB*-qPCR-R | CGCATCACGGCTCGTCATATCC | | |
